# Influenza, *Campylobacter* and *Mycoplasma* Infections, and Hospital Admissions for Guillain-Barré Syndrome, England

**DOI:** 10.3201/eid1212.051032

**Published:** 2006-12

**Authors:** Clarence C. Tam, Sarah J. O’Brien, Laura C. Rodrigues

**Affiliations:** * London School of Hygiene and Tropical Medicine, London, United Kingdom;; † Health Protection Agency, London, United Kingdom;; ‡ University of Manchester, Manchester, United Kingdom

**Keywords:** Guillain-Barré syndrome, *Campylobacter*, influenza, *Mycoplasma pneumoniae*, *Haemophilus influenzae*, cytomegalovirus, Epstein-Barr virus, sequelae, polyradiculoneuropathy, time-series analysis, research

## Abstract

Guillain-Barré syndrome (GBS) is the most common cause of acute flaccid paralysis in polio-free regions. Considerable evidence links *Campylobacter* infection with GBS, but evidence that implicates other pathogens as triggers remains scarce. We conducted a time-series analysis to investigate short-term correlations between weekly laboratory-confirmed reports of putative triggering pathogens and weekly hospitalizations for GBS in England from 1993 through 2002. We found a positive association between the numbers of reports of laboratory-confirmed influenza A in any given week and GBS hospitalizations in the same week. Different pathogens may trigger GBS in persons of different ages; among those <35 years, numbers of weekly GBS hospitalizations were associated with weekly *Campylobacter* and *Mycoplasma pneumoniae* reports, whereas among those >35 years, positive associations were with influenza. Further studies should estimate the relative contribution of different pathogens to GBS incidence, overall and by age group, and determine whether influenza is a real trigger for GBS or a marker for influenza vaccination.

## Abstract 2

TOC Summary line: *Campylobacter*, *Mycoplasma pneumoniae,* and influenza (or influenza vaccination) act as infectious triggers for Guillain-Barré syndrome.

Guillain-Barré syndrome (GBS) is the most common cause of acute flaccid paralysis in polio-free regions. Estimated incidence in high-income countries is 0.4–4.0 cases per 100,000 population (*1*). Campylobacter jejuni is the most commonly identified infectious trigger for GBS. Several studies have demonstrated evidence of recent C. jejuni infection in a higher proportion of GBS case-patients than in controls (*2**–**10*). Other pathogens, including cytomegalovirus (*7*), Epstein-Barr virus (*7*), Haemophilus influenzae (*11**–**14*), and Mycoplasma pneumoniae (*7**,**15**,**16*), have been suggested as possible GBS triggers, as was influenza vaccination in the United States during 1976–1977 (*17*). However, epidemiologic evidence that implicates these latter agents remains scarce. We conducted a time-series analysis to investigate temporal associations between weekly variations in reports of microbiologically confirmed infections and hospital admissions for GBS.

## Methods

### Reports of Microbiologically Confirmed Infections

Positive microbiologic diagnoses ascertained through voluntary laboratory reporting in England and Wales are recorded in the national infections database (LabBase2) (*18*). We obtained weekly reports of infections suspected of causing GBS, namely, Campylobacter spp., cytomegalovirus, Epstein-Barr virus, Haemophilus influenzae (B and non-B), Mycoplasma pneumoniae, and influenza (A, B, and all influenza) from 1993 through 2002. Influenza vaccination figures are available only quarterly and do not provide sufficient temporal resolution for this analysis.

We used the specimen date for all analyses because onset dates were rarely available. For Campylobacter, the median delay between patients' onset date and the specimen date was 4 days (interquartile range 3–7 days); for 90% of cases, the delay was <14 days (*19*). Similar data were unavailable for other pathogens.

### GBS Hospitalizations

Nonidentifiable GBS hospitalization data were provided by Hospital Episodes Statistics (HES) (*20*), which records all in-patient care episodes in English National Health Service hospitals. An episode is a continuous period of treatment under 1 consultant. Each episode includes patient's age, sex, admission date, episode duration, episode number, and <14 possible International Classification of Diseases (ICD) diagnoses. Because a patient may see several consultants during a hospital stay, several episodes for the same hospitalization event may appear in HES records. Since April 1997 a unique code identifies episodes for the same patient. A series of continuous episodes constitutes a spell of treatment. This method of episode linkage was applied to records from January 1998 onward. Before then, only episodes classified as first episodes were used to avoid including multiple episodes for the same patient spell.

A GBS admission was defined as a spell with GBS-related ICD codes (ICD-9 357.0/ICD-10 G61.0) in any of the first 3 diagnostic codes. From 1998 through 2002, 3,477 repeat spells were excluded; these are unlikely to represent independent events, e.g., the likelihood of recurrent GBS may depend on host genetic factors.

**Figure 1:**
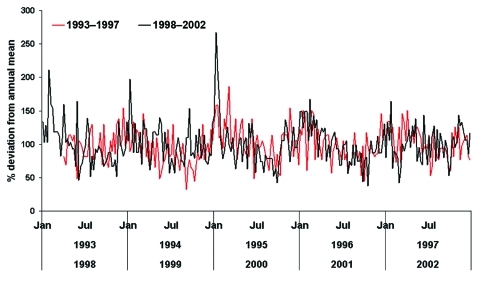
Seasonal distribution of Guillain-Barré syndrome admissions during 1993–1997 and 1998–2002.

The 2 series of hospitalizations (1993–1997 and 1998–2002) were collapsed into weekly counts of GBS admissions. For 1993, data were only available from April 1 on. We compared the 2 periods to investigate whether inability to exclude repeat spells from 1993 through 1997 affected the seasonal pattern of GBS admissions. No major differences were seen (Figure 1), and the 2 periods were combined into a weekly time-series of 10 years.

### Statistical Analysis

We aimed to answer the following question: Is an increase in the number of laboratory reports in any given week associated with increases in GBS hospitalizations in subsequent weeks? In choosing appropriate statistical methods, special characteristics of time-series data must be considered. Such data exhibit nonrandom patterns over time. These include long-term increasing or decreasing trends (whereby weekly GBS hospitalizations within a year are more closely related than between years) and seasonal patterns (whereby the number of GBS hospitalizations in any given week is similar to that in the same week for other years). In addition, weekly hospitalizations are count data, following a Poisson rather than a normal distribution.

For these reasons, time-series observations cannot be considered to be independent, and statistical techniques commonly used for independent, normally distributed data (such as simple correlation) are inappropriate. Special methods that account for temporal dependence in the data are needed. Specifically, temporal dependence in time-series data can result in confounding due to long-term trends (year-on-year) and seasonal (within-year) patterns. Two variables could apparently be related in time because they have similar seasonal characteristics, not because one causes the other. For example, bottled water consumption increases in summer, when the incidence of salmonellosis is highest. This does not imply that bottled water is a risk factor for Salmonella infection; rather, bottled water consumption is influenced by ambient temperature, which itself independently influences Salmonella transmission. The exposure-outcome association could also be confounded or obscured by other time-varying factors. For example, if influenza causes GBS, GBS admissions should increase in winter, when influenza incidence is highest. However, Campylobacter has the opposite seasonality; if Campylobacter is also associated with GBS, high numbers of GBS admissions could still occur when influenza reports are low, because these GBS cases are due to Campylobacter (or other pathogens with different seasonality). Time-series methods account for such temporal dependencies in data by adjusting for these long-term trends and seasonal patterns, which enables associations to be investigated over shorter periods, independent of trend and seasonal components.

We used multivariable Poisson regression adapted for time-series data (*21**–**23*) to investigate the effect of weekly variations in reports of different pathogens on the number of GBS hospitalizations; we adjusted for long-term trends and seasonality. The weekly number of GBS hospitalizations was the outcome, and the weekly number of reports for each pathogen was the exposure. We assumed a log-linear relationship between exposure and outcome, i.e., that an increase in the number of reports of a particular pathogen resulted in a constant increase in the log number of GBS hospitalizations throughout the range of laboratory reports.

We adjusted for long-term (year-on-year) trends by including a variable indicating the year of hospitalization in the regression model; thus, we allowed the mean number of GBS hospitalizations to vary between years. We controlled for seasonality by using Fourier terms (*21**,**24*). Fourier terms can be used to produce a smooth function of expected values for any set of periodic data (e.g., a seasonal pattern). This is achieved by introducing into the regression model a linear combination of pairs of sine and cosine terms (harmonics) of varying wavelengths. A harmonic is an integer fraction of 1 full wavelength (here, 1 year). The more harmonics used, the better the fit to the hospitalization series (i.e., the greater the level of seasonal adjustment). A seasonal pattern with a single peak and single trough within 1 year could be reproduced with 1 harmonic. In reality, seasonal patterns are more complex, and several harmonic terms are required for adequate seasonal adjustment. Given sufficient seasonal adjustment, all variation in the hospitalization series explained by seasonality is removed; any remaining variation must be due to other factors or random noise. This residual variation, independent of long-term trends and seasonal patterns, and its association with weekly reports of infections was our focus of interest. We used 6 harmonics to adjust for periodic patterns in the data >2 months, assuming that GBS risk is increased for <2 months after infection. In addition, we introduced a variable that indicated weeks in which public holidays occurred, to adjust for artifactual variation in laboratory reporting and hospitalizations during these weeks.

### Lag Effects

Because of the time lag between infection and GBS, the number of GBS admissions is likely to be associated not with the number of laboratory reports in the same week, but with the number of reports some time before. We thus performed separate regressions with exposure variables lagged by <8 weeks. Further, because of delays in seeking healthcare, diagnosing infection, and reporting positive diagnoses to national surveillance, increases in hospitalizations could precede increases in laboratory reports. To account for this possibility, we also performed regressions of GBS admissions against laboratory reports within the subsequent 4 weeks.

The core models thus contained the logged GBS hospitalization series as the dependent variable, indicator variables for year, Fourier terms for season, and an indicator variable for weeks with public holidays. The weekly number of reports of a particular pathogen, either in the same week or lagged by a certain number of weeks, was then introduced as the explanatory variable of interest. The regression equation for the models predicting the expectation of the logarithm of weekly GBS hospitalizations, Y, was



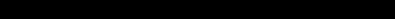



where δ_y_ represents the coefficients for each year (y), S(t) represents a smooth function of season (comprising 6 harmonics), and φ (holiday) is a term representing weeks with public holidays. The regression coefficient, β, is the effect of the exposure of interest (the weekly number of laboratory reports, X). Its exponential, the relative risk (RR), reflects the ratio increase in GBS hospitalizations per unit increase in laboratory reports of pathogen (p) at lag t-l, where l ranges from 8 weeks before to 4 weeks after the GBS hospitalization.

We fit separate models for each pathogen at each lag. We assessed model fit by looking at residual variation. We used the partial autocorrelation function (*25*) to investigate the presence of residual autocorrelation, i.e., whether residual variation in GBS hospitalizations in any given week was correlated with residual variation in other weeks. Some degree of autocorrelation at a lag of 1 week remained after adjustment for yearly and seasonal patterns. We controlled for this by adding to all models a term for the residuals lagged by 1 week (a first-order autoregressive term; AR_1_ in the equation). The scale parameter for standard errors was set as the Pearson χ^2^ statistic divided by the residual degrees of freedom to allow for possible overdispersion in the data.

### Age Group Analysis

**Figure 2:**
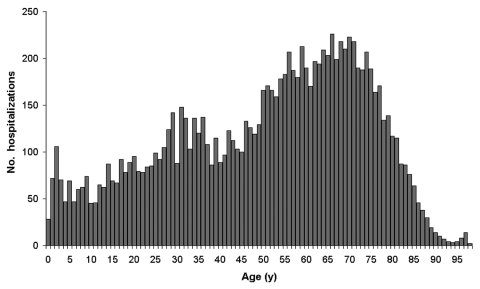
Age distribution of first hospitalization for Guillain-Barré syndrome, England, April 1993–December 2002.

Associations between GBS and infection could differ between age groups; an association between a pathogen and GBS might only become apparent in a limited age range. We performed subanalyses to investigate associations in different age groups. Because the age distribution of GBS case-patients is not uniform, we categorized age into 3 broad groups: <35 years, 35–64 years, and >65 years, according to the age distribution of GBS patient admissions (Figure 2). Age group–specific models were fit similarly to those for all ages.

All lags at which positive associations were found are presented. However, because of the large number of statistical tests performed (13 lags per pathogen per age group), a positive association at a given lag was considered potentially relevant only if it occurred within a cluster. A cluster was defined as 2 or more consecutive lags, each associated with the outcome at the 0.05 significance level. This approach reduces the probability of observing chance associations due to multiple testing. Within clusters, lags significant at the 0.01 level were considered important. The adjusted coefficient from these models was used to calculate the expected increase in GBS admissions per 10% increase in the range of laboratory reports at a given lag. All statistical analyses were performed in Stata 8.0 (Stata Corp., College Station, TX, USA).

## Results

In the 10-year study period, 11,019 primary admissions for GBS occurred: 2,929 (26.6%) patients were <35 years of age, 4,467 (40.5%) were ages 35–64 years, and 3,623 (32.9%) were >65 years. Summary statistics for the weekly number of GBS admissions and laboratory reports for the different pathogens are found in Table 1.

**Table 1:** Summary statistics for weekly number of GBS admissions and laboratory reports, England, 1993–2002*

Condition/pathogen	Mean	SD	Median	25th percentile	75th percentile	Minimum	Maximum	10% range
GBS	21.7	6.7	21	17	26	6	51	4.5
*Campylobacter* spp.	908.2	287.5	875	684	1,104	296	1,737	144.1
Cytomegalovirus	23.5	7.4	22	19	28	6	51	4.5
Epstein-Barr virus	9.9	5.9	10	6	14	0	33	3.3
*Haemophilus influenzae* non-B	9.2	7.0	7	4	12	0	39	3.9
*H. influenzae* B	1.0	1.4	0	0	1	0	8	0.8
*Mycoplasma pneumoniae*	19.6	14.0	15	9	26	1	77	7.6
Influenza	41.4	62.4	13	4	50	0	407	40.7
A	30.9	54.9	9	3	31	0	398	39.8
B	10.4	26.9	2	1	5	0	188	18.8

*GBS, Guillain-Barré syndrome; SD, standard deviation.

Table 2 gives details of the lags for each pathogen for which significant associations with GBS admissions were found. Only clusters of lags that were significant at the 0.05 significance level are presented. Within clusters, lags that were significant at the 0.01 significance level appear in bold. For example, for influenza reports in all ages, significant associations were found between the number of GBS hospitalizations in any given week and the number of influenza reports in the same week (lag 0) and the previous week (lag 1); the p value for the coefficient at lag 0 was <0.01. Lags that were associated with GBS at the 0.05 significance level but did not occur in clusters are shown in Table 3. Seventeen such lags occurred, consistent with 1 in 20 tests giving a significant result by chance (at p<0.05) (given 312 combinations of pathogens, age groups, and lags).

**Table 2:** Poisson regression of Guillain-Barré syndrome (GBS) admissions against infection reports, England, 1993–2002: clusters of lags significant at 0.05 level*†

Pathogen	All ages	<35 y	35–64 y	>65 y
*Campylobacter*	–	3,**4**,**5**	–	–
Influenza	**0**,1	–	R1,0,**1**,**2**	R1,**0**,**1**
A	**0**,1	–	–	R1,**0**,**1**
B	–	–	–	–
*Mycoplasma pneumoniae*	–	R4,**R3**;**R1**,**0**	–	–
*Haemophilus influenzae* non-B	–	–	–	–
*H. influenzae* B	–	–	–	–
Cytomegalovirus	–	–	–	–
Epstein-Barr virus	–	–	–	–

*Regression models are adjusted for yearly trend, seasonal pattern (up to 6th harmonic) and public holidays. †Numbers indicate the lag number in weeks; lag numbers preceded by R represent lags of *n* weeks following the week of admission for GBS. Lags in **boldface** are significant at the 0.01 level of precision.

**Table 3:** Poisson regression of Guillain-Barré syndrome (GBS) admissions against infection reports, England, 1993–2002: unclustered lags*

Pathogen	All ages	<35 y	35–64 y	>65 y
*Campylobacter*	–	7	–	–
Influenza	–	–	R3	–
A	–	–	R1,1	–
B	–	–	2,5	–
*Myplasma pneumoniae*	–	3	–	2
*Haemophilus influenzae* non-B	8	–	7	–
H. *influenzae* B	–	5	–	R2
Cytomegalovirus	–	R3,5	–	6
Epstein-Barr virus	–	R1	5	–

*Lags significant at the 0.05 level of precision but not occurring in clusters (regression models are adjusted for yearly trend, seasonal pattern [up to 6th harmonic] and public holidays).

Table 4 presents RRs and 99% confidence intervals (CIs) for the associations shown in Table 2. Only those individual lags from Table 2 that were significant at the 0.01 level of precision are presented. The RRs represent the relative increase in GBS admissions per 10% increase in the range of laboratory reports for a given pathogen at a given lag. For example, for influenza A, the maximum number of laboratory reports in any given week was 398, while the minimum was zero; an increase in influenza A reports of 39.8 (10% of the range) in any given week results, on average, in a 1.03-fold (or 3%) higher incidence of GBS admissions in the same week (RR = 1.032, 99% CI 1.008–1.057). Overall, a positive association was found only with influenza and influenza A at a lag of zero weeks (in the same week as GBS admission).

**Table 4:** Poisson regression of Guillain-Barré syndrome (GBS) admissions against infection reports, England, 1993–2002: regression coefficients*†

Age group/pathogen	Lag no.	10% range in laboratory reports	RR	99% CI	p value
All ages					
Influenza	0	40.7	1.032	1.008–1.057	0.001
Influenza A	0	39.8	1.029	1.006–1.054	0.001
<35 y					
* Campylobacter*	4	72.0	1.084	1.017–1.156	0.001
	5	72.0	1.074	1.007–1.146	0.004
*Mycoplasma pneumoniae*	R3	5.6	1.040	1.002–1.079	0.007
	R1	5.6	1.043	1.004–1.083	0.004
	0	5.6	1.041	1.002–1.082	0.006
35–64 y					
Influenza	1	16.9	1.051	1.003–1.102	0.006
	2	16.9	1.047	0.999–1.097	0.011
>65 y					
Influenza	0	14.8	1.074	1.024–1.126	0.000
	1	14.8	1.051	1.001–1.104	0.008
Influenza A	0	14.3	1.075	1.027–1.126	0.000
	1	14.3	1.052	1.004–1.103	0.005

*Relative risks (RRs) and 99% confidence intervals (CIs) for significant lags by age group and pathogen. †RRs represent the relative increase in GBS admissions for every 10% increase in the range of laboratory reports for a given pathogen at a given lag.

Different pathogens are associated with GBS admission in different age groups. In those <35 years, the number of GBS admissions in a given week was associated with the number of Campylobacter spp. reports 5 and 4 weeks earlier and with the number of M. pneumoniae reports in the same week and 1 and 3 weeks later.

Among persons ages 35–64 years, a positive association was found between the number of GBS admissions in any given week and the number of all influenza reports 1 and 2 weeks earlier. In those ages >65 years, associations were found between the number of GBS admissions and the number of all influenza and influenza A reports in the current week and 1 week before hospitalization.

The results were robust to varying degrees of seasonal adjustment; we repeated the analysis and adjusted for seasonal wavelengths of up to 4 months (3 harmonics) and 1 month (12 harmonics) and used indicator variables for month as well, all with similar results. Table 5 shows those lags that consistently appeared in clusters at all levels of seasonal adjustment. Results for influenza and M. pneumoniae were not sensitive to the degree of seasonal adjustment. For Campylobacter, clusters of lags were seen with all Fourier models, but not with models that used month indicators.

**Table 5:** Poisson regression of Guillain-Barré (GBS) syndrome admissions against infection reports, England, 1993–2002: varying seasonal adjustment*†

Pathogen	All ages	<35 y	35–64 y	>65 y
*Campylobacter*	–	–	–	–
Influenza	**0**,1	–	**R1**,0,**1,2**	R1,**0**,1
A	**0**,1	–	–	R1,**0,1**
B	–	–	–	–
*Mycoplasma pneumoniae*	–	R4,**R3; R1,0**	–	–
*Haemophilus. influenzae* non-B	–	–	–	–
H. *influenzae* B	–	–	–	–
Cytomegalovirus	–	–	–	–
Epstein-Barr virus	–	–	–	–

*Significant lags consistently found in clusters with all forms of seasonal adjustment (3, 6, and 12 harmonics and month indicators); regression models are additional adjusted for yearly trend and public holidays. †Only clusters of lags significant at the 0.05 level of precision are presented. Numbers indicate the lag number in weeks; lag numbers preceded by R represent lags of *n* weeks following the week of admission for GBS. Lags in **boldface** are significant at the 0.01 level of precision.

## Discussion

We found associations between the weekly number of laboratory reports of various pathogens and incidence of GBS hospitalizations. Different organisms may be responsible for triggering GBS in different age groups. In particular, Campylobacter and M. pneumoniae appear to be associated with GBS in those <35 years, while influenza associations were seen in those >35 years. Differences in the pathogens responsible for triggering GBS in different age groups have not previously been reported.

No clusters of significant lags were found for cytomegalovirus, Epstein-Barr virus, and H. influenzae infections. This could be due to low statistical power (on average, <10 reports per week were made for Epstein-Barr virus and H. influenzae), or it could indicate a very small risk or none after these infections.

Our results are subject to several limitations. HES data exclude information from private hospitals. Given the universality of healthcare in England, however, the proportion of GBS cases diagnosed in private hospitals is likely to be small. Although we used only ICD codes specific for GBS, several GBS cases may be classified under nonspecific codes, namely, ICD-9 357.9 (unspecified toxic and inflammatory neuropathy) and ICD-10 G61.9 (inflammatory polyneuropathy unspecified). However, these codes include a large proportion of cases unrelated to GBS; their inclusion in the analysis would dilute any associations with the infections investigated. Misdiagnosis of GBS is also possible; a proportion of GBS diagnoses is likely to represent false positives. We could not validate diagnoses through hospital chart review because identities were hidden in the HES data, and we did not have access to patients' records. However, any misclassification arising from inclusion of false-positive GBS diagnoses will be nondifferential, i.e., the likelihood of misdiagnosis with GBS is unrelated to the likelihood of diagnosis with the pathogens investigated. Inclusion of non-GBS cases could have resulted in effect dilution, but this inclusion would likely not have yielded positive associations when none truly existed.

Laboratory reports for any condition represent only a subset of all symptomatic cases of disease in the community. Our analysis assumes that, for a given condition, the seasonal pattern of laboratory reports accurately reflects the pattern of all community cases. Ascertainment of influenza is likely to be more comprehensive in winter because microbiologic investigation for this pathogen is not routinely conducted outside the influenza season (*26*). This could affect our ability to detect associations in different seasons, but this was not the focus of our study. Our analysis also assumes that the seasonal pattern of laboratory reports is accurately reflected within each age group. This may not be true if, for example, younger persons are less likely to visit the health services (and, thus, be included in laboratory reports) for symptoms of influenza during the influenza season. However, laboratory report data for influenza show a distinct and consistent peak during the winter months in all age groups (data not shown).

Among Campylobacter spp., only C. jejuni is thought to cause GBS. As clinical isolates of Campylobacter are not routinely speciated in England and Wales, non-jejuni species could not be excluded from the analysis. However, the England and Wales Campylobacter Sentinel Surveillance Scheme indicates that 80%–90% of reports of Campylobacter infection are due to C. jejuni (*19*); inclusion of species not linked to GBS would attenuate rather than inflate any effect on GBS admissions.

The regression coefficients indicate associations between the incidence of various pathogens and GBS admissions, but the coefficients themselves are not directly comparable between pathogens. This is because their magnitude is dependent not only on the true magnitude of the association, but also on the proportion of all cases that is captured by laboratory reports, and this will vary by pathogen (for example, severe conditions are more likely to be reported). Thus, estimates of the relative incidence of GBS due to the different pathogens cannot be obtained from these data. In addition, some evidence exists, particularly for C. jejuni, that GBS can develop after subclinical infection. Our analysis did not include asymptomatic infections, so our results apply only to clinical cases of infection. These findings nevertheless raise hypotheses that merit further investigation. For example, several case reports and immunologic analyses have suggested a link between M. pneumoniae infection and GBS (*7**,**15**,**16*), but such a link has not been confirmed by robust epidemiologic studies. Our results suggest that studies focusing on younger GBS patients could help clarify any such association.

Whether the associations with influenza are real or whether they reflect seasonal patterns in influenza vaccination is unclear. Influenza vaccination has previously been linked to GBS. During the mass vaccination campaign against swine influenza in the United States during 1976–1977, GBS incidence among vaccinees was 7-fold higher in the 6 weeks after vaccination than in nonvaccinees (*17**,**27*). Similar analyses during subsequent influenza seasons (with no mass vaccination) have found no increased risk (*28*), or a doubling of the risk (*29*), which suggests that differences in antigenic formulation or characteristics of vaccinated populations are influential factors in vaccine-related GBS risk. Here, we found associations with influenza A only; this may reflect the antigenic composition of influenza vaccines, or differential risk resulting from antigenic differences between subtype A and B strains of influenza.

The short lags identified here between increases in influenza reports and subsequent GBS admissions are consistent with a vaccine trigger; the risk period for vaccine-related GBS is believed to be 6 weeks, and increases in vaccination coverage would be expected to precede seasonal rises in influenza. Vaccination could also explain the lack of an association in younger persons, because influenza vaccination is not generally recommended in healthy persons <65 years in the United Kingdom. Influenza vaccine coverage data indicate that for the study period, vaccine uptake was <1% in low-risk groups ages <35 years, <10% among those ages 35–54 years, and 20%–30% among those ages >65 years (*30*). For elderly persons at high risk, uptake increased from 40% to 65% from 1993 through 2002 (*31*). These data support the hypothesis that persons in older age groups have a greater vaccine-induced risk of GBS, although a true association with the disease of influenza is still possible. Primary care–based studies investigating the influenza and influenza vaccination status of GBS patients could help resolve this issue.
